# Concurrent Warthin tumor and Kimura disease: a case report

**DOI:** 10.1186/s13256-022-03729-5

**Published:** 2023-01-08

**Authors:** Asma Almazyad, Naheel Al Khudiri, Saeed S. Alshieban, Majed M. Pharaon

**Affiliations:** 1 King Saud Bin Abdulaziz University for Health Sciences, P.O. Box 3660, Riyadh, 11481 Saudi Arabia; 2 King Abdullah International Medical Research Center, P.O. Box 3660, Riyadh, 11481 Saudi Arabia; 3 Department of Pathology and Laboratory Medicine, King Abdulaziz Medical City, Ministry of National Guard Health Affair, P.O. Box 22490, Riyadh, 11426 Saudi Arabia

**Keywords:** Hybrid lesions, Warthin tumor, Kimura disease, IgE, Case report

## Abstract

**Background:**

Warthin tumor (WT) is a common benign salivary tumor of the parotid gland. Clinically, it occurs in men in their fifth to seventh decades who typically smoke cigarettes. WTs have been reported with different head and neck neoplasms and other salivary gland tumors within the same or another salivary gland. Kimura disease (KD) is a rare chronic inflammatory disease with unknown etiology affecting young to middle-aged Asian men. KD presents as an asymptomatic nodule in the head and neck area, with regional lymphadenopathy and salivary gland involvement.

**Case presentation:**

A 64-year-old Arabic man presented with a 10-year history of an asymptomatic swelling of the left face. Computed tomography showed a well-defined, multicystic mass with heterogeneous enhancement. The resected mass was composed of two distinct components. There was a well-demarcated proliferation of papillary and cystic oncocytic epithelium with lymphoid stroma, consistent with WT. Some areas exhibited sclerotic fibrosis, with multiple lymphoid follicles showing folliculolysis, follicular hyperplasia, and eosinophilic infiltrate. The patient’s immunoglobulin E level serum was elevated, confirming a coexisting KD. The patient underwent a left superficial parotidectomy, with no recurrence at a 30-month follow-up.

**Conclusion:**

This report describes the first concurrent case of WT and KD in the parotid gland.

## Background

Warthin tumor (WT) is the second most common benign salivary gland tumor, accounting for 12–37% of parotid gland neoplasms [1, 2]. WT is believed to arise from salivary gland remnants that reside within the intra-parotid lymph nodes [3]. Typically, it presents in the fifth to seventh decades of life, with historically strong male predilection; currently, there is an increase in the incidence of WT in females. There is a positive association between WT and cigarette smoking, where almost 90% of patients with WT have a history of smoking [4]. Most cases occur in the superficial lobe of the parotid gland, with bilateral or multifocal presentation seen in 10% and 12%, respectively [5]. Fine needle aspiration (FNA) is the primary diagnostic procedure for WT, where it demonstrates sheets of lymphocytes admixed with oncocytic epithelial cells [6]. WT has pathognomonic histological features; it consists of papillary cystic spaces lined by a double layer of oncocytic cells and lymphoid tissue within the stroma [1].

Kimura disease (KD) is a rare chronic inflammatory disorder that occurs predominantly in young Asian males, with sporadic cases reported in other regions [7]. It presents as a painless subcutaneous soft nodule, most commonly in the head and neck area, with regional lymphadenopathy and occasional salivary gland involvement [7]. Patients with KD show peripheral eosinophilia and elevated serum immunoglobulin E (IgE) levels. Although KD mimics neoplasm clinically, it is a reactive process, and the pathogenesis remains unclear. Histologically, KD has three main components: densely collagenous stroma, florid germinal center hyperplasia with abundant eosinophils, and postcapillary venule proliferation [8].

Coexistence of two different entities in a patient is an uncommon phenomenon. However, the presence of other non-salivary gland neoplasms with the very common WT has been reported to be as high as 37%, with head and neck squamous cell carcinoma being the most common neoplasm [9]. Synchronous salivary gland neoplasms are seen most commonly with WT and usually occur bilaterally [10]. Only one rare case of parotid WT coexisting with Langerhans cell histiocytosis (LCH) was reported in the same gland [11]. In this report, we describe a case of concurrent WT and KD in the parotid gland, the first that we are aware of in the English literature, and describe the clinicopathological features of the case.

## Case presentation

**Fig. 1:**
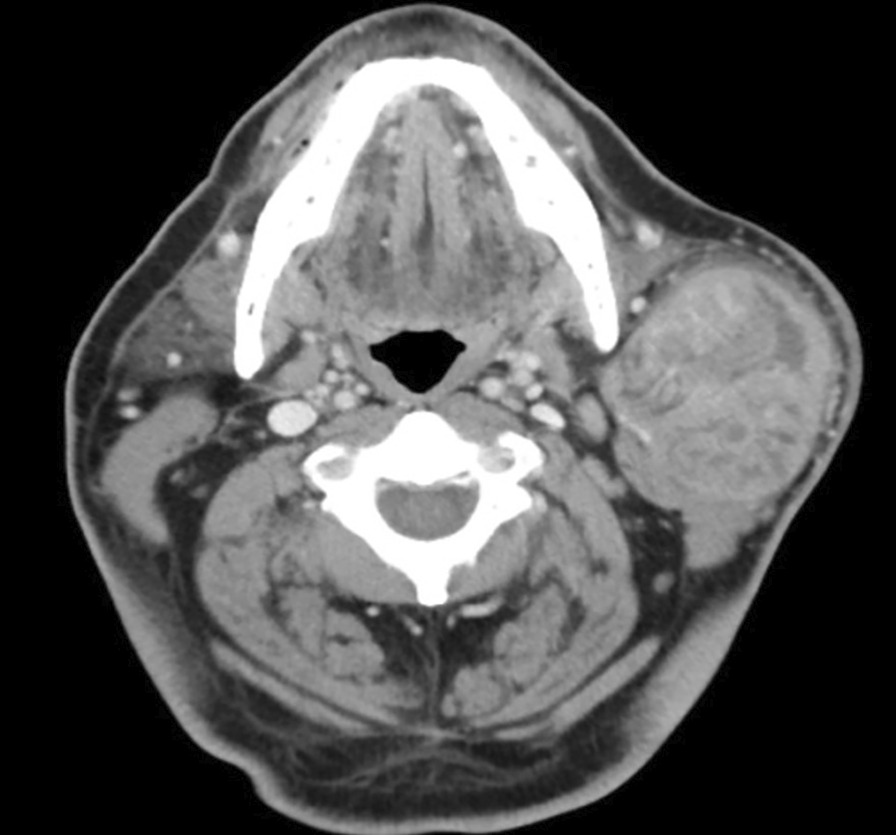
Computed tomography scan, coronal view: well-defined nodule with heterogeneous enhancement and multiple cystic spaces in the left parotid gland

A 64-year-old Arabic man presented with a left painless swelling of the face of a 10-year duration. The patient reported a recent increase in the size of the swelling. His medical history was significant for hypertension and cigarette smoking. Head and neck examination revealed a firm, non-tender parotid mass, measuring 10 cm in the greatest dimension. There was limited mobility of the mass, and the overlying skin exhibited no changes. There were no lymph nodes or facial nerve abnormalities. The computed tomography scan showed a 7.1 × 6.2 × 5.5 cm well-defined mass in the left parotid with heterogeneous enhancement and multiple cystic spaces. The mass displaced the sternocleidomastoid muscle posteriorly and medically but showed no infiltration (Fig. 1). FNA was performed at an outside facility and was reported as WT; the slides were not available for an in-house evaluation. The patient underwent a left superficial parotidectomy at our facility. Interestingly, the surgeon commented on the tumor consistency being firmer than a typical WT.

**Fig. 2:**
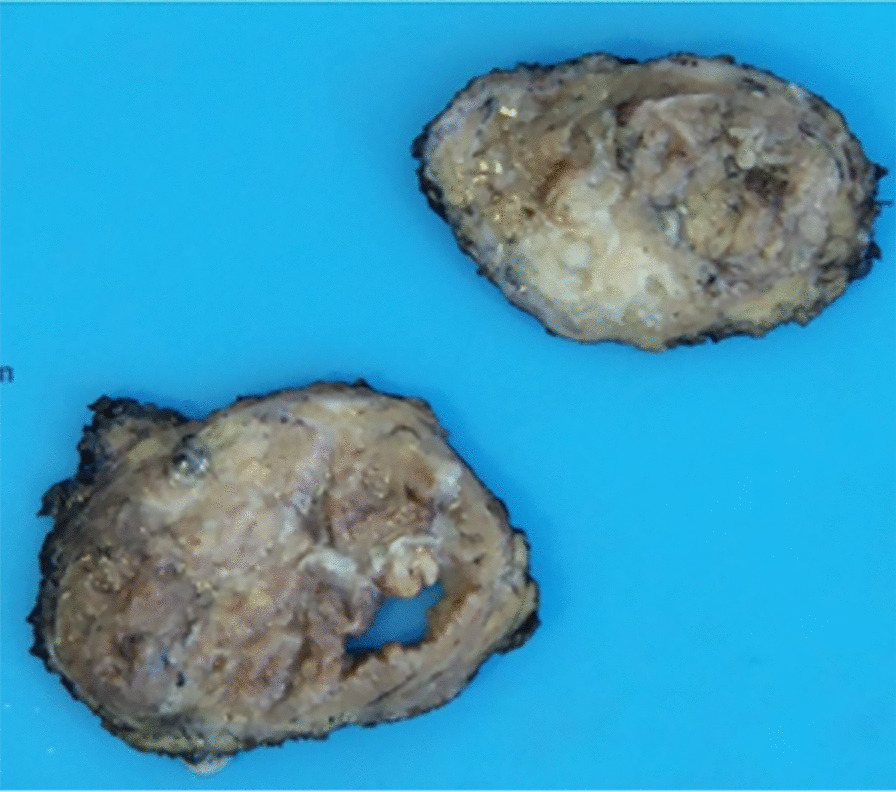
The gross specimen consisted of a tan-brown solid mass with a well-circumscribed border and cystic spaces

**Fig. 3:**
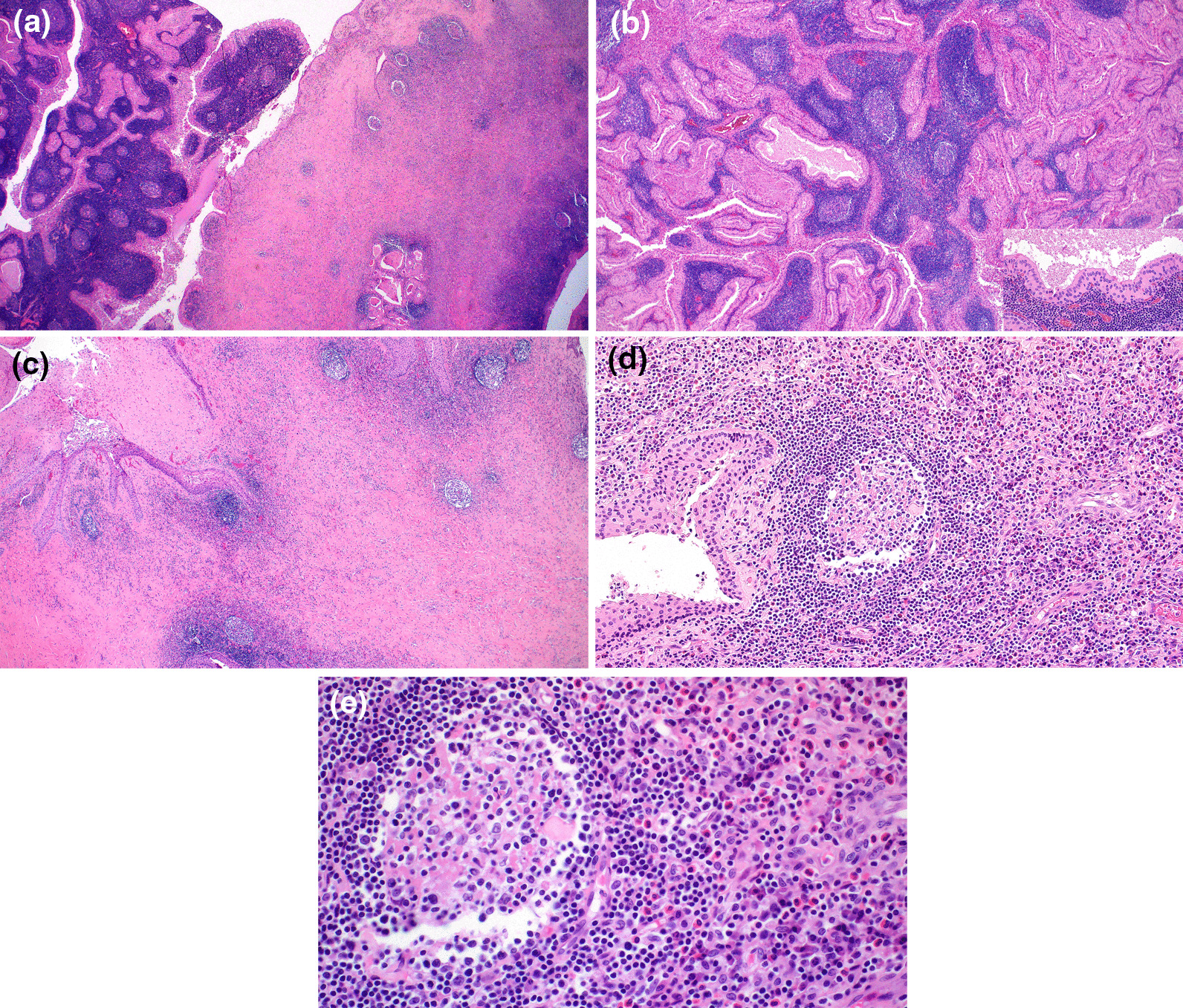
**A** The mass consisted of two main components: multiple papillary growths and cystic spaces with bright eosinophilic cells within lymphoid tissues (left) and extensively fibrotic tissue with multiple vaguely formed lymphoid follicles (right). (H&E, original magnification ×20). **B** The first component consisted of multiple papillary cystic spaces lined by oncocytic epithelium with diffuse lymphocytic infiltrate with lymphoid follicle in the stroma. (H&E, original magnification ×40) (Inset: the cystic lining is bilayer of luminal columnar cells and abluminal cuboidal cells; both cells have granular eosinophilic cytoplasm. (H&E, original magnification ×200). **C** The second component of the mass showed diffuse sclerotic fibrosis with scattered lymphoid follicles exhibiting folliculolysis, and inflammatory infiltrate. (H&E, original magnification ×40). **D** A prominent eosinophilic infiltrate was present within the sclerotic stroma and between the lymphoid follicles, which were admixed with lymphocytes and histiocytes. (H&E, original magnification ×100). **E** The lymphoid follicles showed follicular hyperplasia with mantle zone preservation with proteinaceous deposits and perivascular fibrosis. (H&E, original magnification ×200)

Gross examination of the resected parotid tissue showed an 8 cm firm tumor with a yellow to tan cut surface, exhibiting solid and cystic areas (Fig. 2). Histological examination revealed that the nodule had two different components (Fig. 3A). One area consisted of multiple cystic spaces with papillary architecture lined by a double layer of oncocytic cells and surrounded by lymphocytes forming lymphoid follicles with germinal centers. Cellular debris and thick secretion within the cystic lumina were observed (Fig. 3B). Consistent with the previous FNA site, there were foci of fibrosis, squamous metaplasia, necrosis, and lymphoplasmacytic infiltrate. In other areas of the tumor, there was a prominent eosinophilic infiltrate admixed with lymphocytes and histiocytes in a densely fibrotic stroma. There were lymphoid follicles scattered in the fibrotic stroma exhibiting folliculolysis, follicular hyperplasia, eosinophilic deposits, and perivascular sclerosis (Fig. 3C–E).

The follicular dendritic cell meshwork in the background of the lymphoid follicles was highlighted with CD21 and CD23. To exclude histiocytic neoplasms, CD1a and S100 were done and showed no positivity within the tumor cells (data not shown). A complete blood count with differential and serum IgE level were performed and showed a normal absolute eosinophil count of 0.41 × 10^9^/L and elevated serum IgE level at 2691 KU/L. Based on the histological features and laboratory test results, a diagnosis of KD coexisting with WT was rendered. There was no evidence of recurrence at a thirty-month follow-up.

## Discussion

Benign salivary gland tumors account for 73–86% of all parotid neoplasms, and WT is the second most common tumor (3–35%) after pleomorphic adenoma (73–80%) [12–14]. WT, also known as papillary cystadenoma lymphomatosum, is an oncocytic epithelial neoplasm originally reported by Hildebrand in 1895 and first reported in the English literature by Warthin in 1929 [15, 16]. Clinically, WT presents as a slowly growing mass of the parotid gland in patients mainly in their fifth to seventh decades of life. There is a slight male predilection, with a ratio of 1.6:1 [4, 17]; the gender ratio is less than initially reported (5:1 or 10:1) [18, 19]. The shift in WT incidence in females is likely due to the increase in smoking habits among females, since the majority (90%) of patients with WT have a history of cigarette smoking [4, 5]. WT most commonly occurs in the parotid. Extraparotid WT is infrequently seen in the submandibular gland (1–6.9%) and extremely rarely in the minor salivary glands (0.1–1.2%) [17, 20]. Bilateral or multifocal presentations are not unusual and occur in 10% and 12% of patients, respectively [5]. The exact pathogenesis has been debated, with multiple proposed theories, hence the different terminology used in the literature [21]. It was first thought to be a variant of lateral neck cyst when Hildebrand reported it [15]. However, the most plausible pathogenesis is that WT arises from salivary gland tissue entrapped within the intra-parotid lymph nodes during development. Recently, it was elegantly demonstrated with CAM5.2 reactivity of the extrafollicular reticulum cells within the lymphoid tissue of WTs, confirming that these tumors arise within the lymph nodes [3]. Histological features of WT are straightforward and readily recognizable. It consists of a variable degree of cystic spaces lined by oncocytic epithelium with papillary configuration within lymphoid tissue with occasional germinal centers. A closer examination of the oncocytic lining shows a double layer of luminal columnar cells and abluminal round to cuboidal cells; both cells have granular eosinophilic cytoplasm [1]. WT is treated by superficial or total parotidectomy, with a low recurrence rate of 0.8–4.2% [4].

Synchronous salivary gland neoplasms in the parotid gland are uncommon and account for only 3.6% of all parotid gland neoplasms [10, 22]. The very common WT is reported with almost every benign and malignant salivary gland neoplasm, including pleomorphic adenoma, basal cell adenoma, oncocytoma, mucoepidermoid carcinoma, adenoid cystic carcinoma, salivary duct carcinoma, and acinic cell carcinoma [10, 23–25]. Nonetheless, coexisting non-salivary gland neoplasms with WT are not reported in the literature within the same parotid gland except for one case of WT with LCH. Tan *et al.* [11] reported a synchronous WT and LCH in a 50-year-old man, which presented as painless right parotid swelling with a sudden increase in size and a recent inguinal lump. His blood count showed leukocytosis and eosinophilia. Typical histological features of WT were present. In addition, there were abundant eosinophils with scattered mononuclear cells with convoluted nuclei and distinct nuclear grooves within the intra-parotid lymph nodes; these cells were positive for CD1a and S100, confirming LCH diagnosis. Interestingly, the patient’s tumor also exhibited necrosis, eosinophilic abscesses, and granulomatous reaction within the intra-parotid lymph nodes, which raised KD as a differential diagnosis. However, there was no significant fibrosis, follicular hyperplasia, or other characteristic features of KD [11]. The patient underwent parotidectomy, with no evidence of recurrence after 4 months of follow-up [11].

KD is a rare immune-mediated, inflammatory disorder with unclear etiology. It was initially reported in the Chinese population in 1937 by Kimm *et al.* [26] and in a Japanese population in 1948 by Kimura *et al.* [27]. It presents as a subcutaneous nodule with head and neck predilection in young Asian males in their second to third decades of life [7]. Patients with KD usually have regional lymphadenopathy and/or salivary gland involvement. The majority of KD patients are of Asian descent with sporadic cases in Caucasians, Hispanics, Blacks, and Arabs with a very low incidence of salivary gland involvement [7]. Laboratory test shows peripheral eosinophilia and elevated serum IgE levels, which may have prognostic implications [28]. The main histological features of KD consist of sclerotic fibrosis, prominent eosinophilic microabscesses, follicular hyperplasia, foliculolysis, and postcapillary venule proliferation of the germinal centers. Other features seen in KD include germinal center necrosis, proteinaceous deposits or eosinophilic infiltrates in the germinal centers, polykaryocytes within the germinal center or interfollicular areas, reticular IgE deposition within the germinal center, and perivenular sclerosis [7]. Treatment of KD includes regional or systemic corticosteroids, cytotoxic drugs, or radiation; however, surgical removal is preferred [29].

LCH, Hodgkin lymphoma, and angiolymphoid hyperplasia with eosinophilia (ALHA) were considered in the differential diagnosis in the present case. Immunohistochemical staining of CD1a and S100 failed to detect Langerhans cells. The clinical presentation and the diffuse lymphocytic infiltrate histologically raised the possibility of Hodgkin lymphoma; however, no typical Reed–Sternberg cells were present in the sections examined. Lastly, ALHA was previously confused with KD and used interchangeably in the old literature. ALHA is currently considered a vascular neoplasm with known FOS and FOSB gene rearrangements [30]. Histologically, it shares many features with KD but shows prominent solid growth of endothelial proliferation, with epithelioid appearance and central dilated blood vessels. Additionally, patients with ALHA rarely show peripheral eosinophilia or high serum IgE [31]. The precise pathogenesis of this hybrid WT and KD is unknown. Many hypotheses can be proposed to explain their coexistence, including the possibility of collision tumors from the synchronous occurrence of WT and KD at the same location. Another possibility is that WT produced specific cytokines that induced the inflammatory or reactive processes mimicking KD or vice versa.

## Conclusion

This is a report of concurrent WT and KD, a rare and novel presentation of parotid tumors that occurred as a single nodule with a recent increase in size. Interestingly, the surgeon noted a firmer consistency than a typical WT during parotidectomy. Although the management is similar for both entities, it is necessary to identify both components, since KD may require additional treatment and follow-up. In addition, the relationship between WT and KD is unclear and warrants further investigation.

## Data Availability

Not applicable.
